# Mitochondrial Dysfunction and Aging: Insights from the Analysis of Extracellular Vesicles

**DOI:** 10.3390/ijms20040805

**Published:** 2019-02-13

**Authors:** Anna Picca, Flora Guerra, Riccardo Calvani, Cecilia Bucci, Maria Rita Lo Monaco, Anna Rita Bentivoglio, Hélio José Coelho-Júnior, Francesco Landi, Roberto Bernabei, Emanuele Marzetti

**Affiliations:** 1 Università Cattolica del Sacro Cuore, Institute of Internal Medicine and Geriatrics, 00168 Rome, Italy; anna.picca1@gmail.com (A.P.); coelhojunior@hotmail.com.br (H.J.C.-J.); francesco.landi@unicatt.it (F.L.); 2 Fondazione Policlinico Universitario “Agostino Gemelli” IRCSS, 00168 Rome, Italy; mariarita.lomonaco@policlinicogemelli.it (M.R.L.M.); annarita.bentivoglio@unicatt.it (A.R.B.); emanuele.marzetti@policlinicogemelli.it (E.M.); 3 Department of Biological and Environmental Sciences and Technologies, University of Salento, 73100 Lecce, Italy; guerraflora@gmail.com (F.G.); cecilia.bucci@unisalento.it (C.B.); 4 Università Cattolica del Sacro Cuore, Institute of Neurology, 00168 Rome, Italy; 5 Applied Kinesiology Laboratory–LCA, School of Physical Education, University of Campinas, 13.083-851 Campinas-SP, Brazil

**Keywords:** biomarkers, mitophagy, mitochondrial biogenesis, mitochondrial dynamics, mitochondrial quality control, mitochondrial-derived vesicles (MDVs), exosomes, mitochondrial–lysosomal axis

## Abstract

The progressive decline of cell function and integrity, manifesting clinically as increased vulnerability to adverse outcomes and death, is core to biological aging. Mitochondrial dysfunction, oxidative stress, altered intercellular communication (including chronic low-grade inflammation), genomic instability, telomere attrition, loss of proteostasis, altered nutrient sensing, epigenetic alterations, and stem cell exhaustion have been proposed as hallmarks of aging. These “aging pillars” are not mutually exclusive, making the matter intricate and leaving numerous unanswered questions. The characterization of circulating extracellular vesicles (EVs) has recently allowed specific secretory phenotypes associated with aging to be identified. As such, EVs may serve as novel biomarkers for capturing the complexity of aging. Besides the mitochondrial–lysosomal axis, EV trafficking has been proposed as an additional layer in mitochondrial quality control. Indeed, disruption of the mitochondrial–lysosomal axis coupled with abnormal EV secretion may play a role in the pathogenesis of aging and several disease conditions. Here, we discuss (1) the mechanisms of EV generation; (2) the relationship between the mitochondrial–lysosomal axis and EV trafficking in the setting of mitochondrial quality control; and (3) the prospect of using EVs as aging biomarkers and as delivery systems for therapeutics against age-related conditions.

## 1. Introduction

Aging is marked by multiple biological disarrangements that increase the risk of developing several chronic diseases (e.g., cardiovascular disease, diabetes, cancer, and neurodegeneration) and functional decline, which both contribute to negative health-related events (e.g., poor quality of life, morbidity, disability, loss of independence, institutionalization, death) [1,2]. Such a scenario well depicts the urge of untangling the determinants of aging for devising strategies able to extend healthspan and foster active aging.

Nine biological processes have recently been proposed as “aging pillars”: genomic instability, telomere attrition, epigenetic alterations, loss of proteostasis, deregulated nutrient sensing, mitochondrial dysfunction, cellular senescence, stem cell exhaustion, and altered intercellular communication [3].

The garbage catastrophe theory of aging holds that the loss of efficiency of cellular quality control mechanisms, especially autophagy, results in the progressive accumulation of intracellular “waste”, including protein aggregates, damaged mitochondria and lipofuscin, that further depresses cell recycling processes and homeostasis [4]. While extracellular components are delivered and degraded into lysosomes via endocytic pathway, cytosolic materials, including dysfunctional mitochondria, are directed to lysosomes via autophagy, the main intracellular recycling machinery [5]. 

Emerging evidence indicates that in settings of incomplete mitochondrial depolarization, cells can remove unwanted materials and damaged organelles through their release into the extracellular compartment via specialized vesicles [6]. Extracellular vesicles (EVs) can shuttle among cells and the delivery of their cargo can produce beneficial or detrimental effects on neighboring cells, depending on the nature of the transported materials [6]. As such, EVs have been advocated as biomarkers for capturing aging complexity. A great deal of work in preclinical models supports the hypothesis that interventions targeting cellular quality control and EV trafficking may extend health- and lifespan [7,8,9,10,11,12]. A new generation of clinical trials is highly sought after to validate this hypothesis in humans.

## 2. Biogenesis and Characterization of Extracellular Vesicles

EVs were initially considered to be fragments or artifacts of dead and degenerated cells. Subsequently, it was demonstrated that they are mediators of cellular communication with critical roles in cell physiology and pathology [13,14,15].

Although the identification and characterization of EVs are still challenging, they have been distinguished in exosomes and ectosomes based on their biogenesis. In 1991, Stein and Luzio [16] coined the term ectosome referring to structures generated by ectocytosis, the shedding of vesicles from the plasma membrane of stimulated neutrophils. Nowadays, ectosomes are referred to as EVs (diameter 100–500 nm) generated by all cells and identified with various terms: shedding vesicles, microvesicles, exosome-like vesicles, nanoparticles, microparticles, and oncosomes. Exosomes were initially identified as a type of vesicle released from the plasma membrane during the maturation of reticulocytes [17]. The same term has also been used to refer to a molecular machinery involved in RNA processing [18].

Currently, exosomes are referred to as EVs of endosomal origin with a diameter of 50–150 nm. Exosome precursors are intraluminal vesicles (ILVs), generated by the inward budding of discrete domains of the membrane of early endosomes, subsequently evolving into multivesicular bodies (MVBs) [19,20,21,22]. MVBs usually pursue a degradative pathway via their direction to lysosomes. However, under appropriate stimulation, MVBs can move towards the plasma membrane to undergo exocytic fusion followed by release of their ILVs (i.e., exosomes) into the extracellular space [19,20].

A molecular machinery, named endosomal sorting complex required for transport (ESCRT), is activated during local membrane remodeling (e.g., viral budding, cytokinesis, autophagy) [23,24] and is involved in MVBs biogenesis [23,24]. Intracellular translocation and fusion of MVBs are under the control of several G proteins [e.g., Ras Like-1 (RAL1), Ras-related protein in Brain (RAB)27A and RAB27B] [25,26]. In particular, RAB7A is a key regulator of late endocytic traffic [27] that controls exosome production in melanoma cell lines [28], human breast adenocarcinoma MCF7 cells [29], ovarian cancer cells under hypoxic conditions [30], and chemoresistant cervical cell lines [31]. Once released, EVs navigate the extracellular space and reach target cells to regulate physiological (e.g., organismal development [32], immune response [33], neuronal communication [34], tissue repair [35]) or pathological processes (e.g., tumor [36] and neurodegenerative disease progression [37]).

EV cargoes include proteins [38,39], RNAs (mRNAs, miRNAs, and non-coding RNAs) [40,41], and short DNA sequences [38] that are relevant for the maintenance of cell homeostasis and survival, regulation of cell functions, and intercellular communication [42,43]. EVs can activate signaling pathways in target cells also upon their binding to plasma membrane or interaction with intracellular enzymes and factors [20,44]. Furthermore, EVs can regulate gene expression by modulating the activity of transcription factors, signaling proteins and enzymes [20]. Finally, stem cells can generate EVs to favor angiogenesis and organ regeneration [45].

A poorly investigated aspect of EVs pertains the transport of mitochondria or their components. Mitochondrial mtDNA (mtDNA) fragments have been identified within the exosomal cargo in astrocytes and myoblasts [46,47]. Furthermore, larger vesicles (70–150 nm) containing mitochondrial particles in addition to mtDNA are released by mesenchymal stem cells and astrocytes in response to oxidative stress [48,49]. Though, the role of mitochondrial components outside the cells and the mechanisms of their loading into vesicles are not yet completely deciphered. Indeed, mtDNA may be released in the blood independent of its incorporation into EVs [50].

Recently, Sansone et al. [51] identified a full mitochondrial genome in circulating EVs from patients with hormonal therapy-resistant metastatic breast cancer. The authors proposed a role for mtDNA horizontal transfer in mediating cancer resistance to therapy. Indeed, acquisition of EV-mediated mtDNA, especially in cancer stem-like cells, was associated with restoration of oxidative phosphorylation. Furthermore, the cancer cells most capable of producing EVs containing packaged mtDNA were those able to quickly reprogram their metabolism in response to oxidative stress, which contributed to endocrine therapy resistance [51]. 

Much less is known about exosome signaling in the context of aging. Only a few studies have characterized the secretory pathways of senescent cells and found specific senescence-associated secretory phenotypes (SASPs) [52]. However, due to the pivotal role of mitochondrial dysfunction in the aging process, the chase for mitochondrial components within EVs may hold promise for the identification of novel biomarkers for age-related phenomena.

In the next paragraph, we discuss major mechanisms involved in preserving mitochondrial homeostasis and preventing their impairment during aging. A special focus is dedicated to the emerging role of the mitochondrial–lysosomal axis in the generation of EVs for disposing dysfunctional organelles.

## 3. The Emerging Role of the Mitochondrial–Lysosomal Axis in Mitochondrial Quality Control

Mitochondria make up about one fifth of cell volume. These organelles are crucial modulators of cell metabolism and control several other activities (e.g., calcium and iron buffering, iron–sulfur cluster and heme biosynthesis, and programmed cell death) [53]. 

Mitochondrial biogenesis is the process that ensures the number of organelles meets cellular energy demands and is accomplished via coordinated nucleus–mitochondrion crosstalk. Mitochondrial mass decreases during aging as a consequence of reduced mitochondriogenesis [54,55,56]. However, mitochondrial replenishment would not be as efficient if damaged organelles are not adequately disposed [57]. Appropriate mitochondrial quality control (MQC) processes (i.e., mitochondrial proteostasis, dynamics, and autophagy) are therefore in place to guarantee organelle homeostasis. 

Mitochondria are plastic organelles undergoing continuous cycles of fusion and fission that preserve their shape and dilute damage along the network [58]. Besides this, functional mitochondrial dynamics prepare organelles for disposal through their segregation from the mitochondrial network [58]. Severely damaged mitochondria are fissioned and cleared through mitophagy, a highly selective form of autophagy [59]. When operating at its full capacity, this process allows mitochondrial quality within the cell to be maintained. Instead, a decline in MQC efficiency occurs during aging and in the setting of chronic degenerative conditions [3].

Albeit long envisioned as standalone organelles, mitochondria are not floating entities within cytoplasm; rather, they are organized in a dynamic network with several cellular compartments via membrane contact sites and tethering molecules. In particular, connections between the endoplasmic reticulum and mitochondria regulate a number of processes, including calcium homeostasis, lipid trafficking, mitochondrial morphology, cell death, immune response, and autophagosome formation [60,61,62,63]. Furthermore, the interplay between mitochondria and peroxisomes via mitochondrial delivery systems modulates metabolic and redox signaling pathways [64]. 

Recently, functional connections between lysosomes and mitochondria have also been described [65]. Indeed, defects in one of the two organelles are able to induce impairments in the other, thereby suggesting the existence of a mitochondrial–lysosomal axis [66]. For instance, ablation of the mitochondrial transcription factor A (TFAM), which is involved in mitochondrial replication, transcription and maintenance [67], increases the number of lysosomes in T cells [66]. However, lysosomal activity is impaired as a consequence of deficient mitochondrial respiration and the disruption of endolysosomal trafficking. This culminates in the accumulation of sphingomyelin and autophagy intermediates, eventually triggering an inflammatory response [66].

Genetic ablation or pharmacological inhibition of apoptosis inducing factor (AIF), optic atrophy-1 (OPA1) or phosphatase and tensin homolog (PTEN)-induced putative kinase 1 (PINK1) in neurons alters lysosome morphology and activity, thereby inducing accumulation of autophagic substrates [68]. More recently, activation of the lysosomal mechanistic target of rapamycin complex 1 (mTORC1) has been reported to couple nutrient availability to mitochondrial activity and mtDNA replication in neurons, a process termed nutrient-induced mitochondrial activity (NiMA) [69]. Amyloid-β oligomers (AβOs), the precursors of amyloid plaques, block insulin- and amino acid-regulated mitochondrial function, which stimulates mTORC1 activity at the plasma membrane but not at the lysosomal surface followed by cell cycle re-entry and neuronal death. AβOs halt NiMA also in a tau-dependent manner, since tau protein is essential for selective activation of mTORC1 at the plasma membrane by AβOs [70]. These two parallel and interconnected signaling routes initiated by AβOs eventually result in mitochondrial dysfunction, which contributes to neuronal loss in Alzheimer’s disease (AD) [69]. 

Furthermore, restoration of lysosomal acidity using lysosome-targeted nanoparticles has been shown to reinstate mitophagy in pancreatic β cells exposed to high concentrations of free fatty acids [71]. This indicates that, at least under lipotoxic conditions, mitochondrial dysfunction is downstream of lysosomal alkalization and that recovery of lysosomal acidity restores MQC [71].

Mitochondrial-derived vesicles (MDVs) have been proposed as an additional means through which organelle components can be delivered to lysosomes for MQC [72]. The presence of mitochondrial constituents within exosomes is an indirect evidence of crosstalk between mitochondria and the endolysosomal system [51]. This shuttle system does not require mitochondrial depolarization, autophagy signaling or mitochondrial fission [72]. Indeed, MDVs are generated also in cells lacking autophagy-related gene (Atg) 5, Beclin-1 or Rab9 as well as after silencing of dynamin-related protein 1 (DRP1) [72]. As a whole, these findings indicate that MDV delivery to lysosomes for degradation complements mitophagy for MQC.

**Figure 1:**
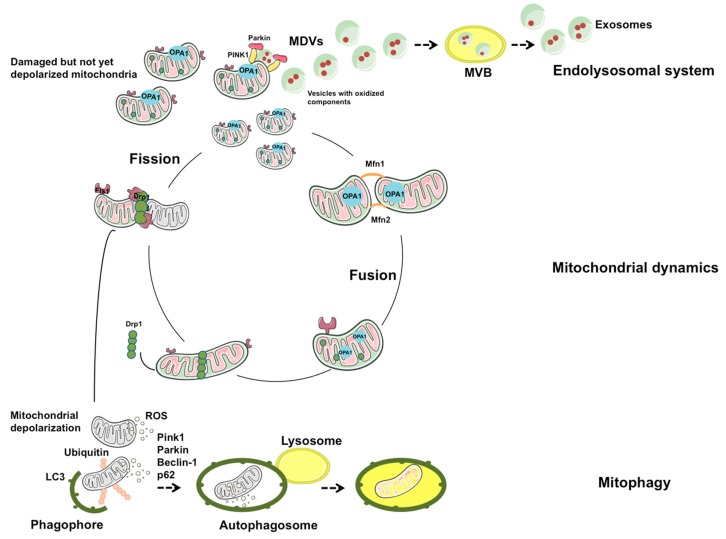
Mitochondrial quality control through mitophagy and mitochondrial-derived vesicle trafficking. Several interrelated processes ensure mitochondrial quality and turnover. Mitochondrial dynamics are mediated by a number of factors that regulate fusion [mitofusin 1 (MFN1), MFN2, and optic atrophy 1 (OPA1)] and fission [dynamin-related protein 1 (DRP1) and mitochondrial fission 1 protein (FIS1)] processes. Fusion facilitates the dilution of damaged mitochondria along the network, while fission targets dysfunctional organelles for their subsequent clearance through mitophagy. Mildly damaged, not yet depolarized mitochondria may be primed by phosphatase and tensin homolog (PTEN)-induced putative kinase 1 (PINK1) and Parkin to generate mitochondrial-derived vesicles (MDVs). MDVs reach out the endolysosomal system forming multivesicular bodies (MVBs) and are released into the extracellular compartment as exosomes. LC3, microtubule-associated proteins 1A/1B light chain 3; ROS, reactive oxygen species.

A fine-tuned coordination between mitophagy and MDV generation has been hypothesized [6]. According to this view, mitophagy would represent an extreme attempt to maintain cell homeostasis through degradation of dysfunctional mitochondria, while MDV generation would be an alternative route to dispose mitochondrial components, before whole-sale organelle degradation is triggered. The fate of a damaged mitochondrion would, therefore, depend on the degree of organelle dysfunction. Indeed, lysosomal delivery of MDV seems to occur as an early response to oxidative stress [73,74]. Conversely, severely damaged mitochondria releasing pro-apoptotic factors and oxidants are fissioned and targeted for elimination through mitophagy [59,75] (Figure 1). Albeit the mechanisms of MDVs generation are unclear, their biogenesis seems to proceed independently of DRP1 and to require priming of PINK1 and Parkin [67]. Via this route, large double-membrane vesicles enriched with mitochondrial matrix cargoes are released. Notably, PINK1 and Parkin represent a point of convergence for MDVs generation and mitophagy. Indeed, MDV extrusion into the extracellular milieu has been proposed as an additional layer of protection when mitophagy is compromised or overwhelmed [76]. 

Although largely indirect, the available evidence suggests that mitochondrial dysfunction during aging may primarily arise from altered MDV trafficking and defective mitophagy. Unveiling the mechanisms of MQC failure is therefore actively sought after to identify novel biological targets for anti-aging interventions.

## 4. Extracellular Vesicle Trafficking and Aging

In vitro and animal studies suggest that age-related intracellular accumulation of noxious macromolecules and organelles is primarily caused by a loss of efficiency of degradative pathways [3]. Initial evidence indicates that quality control processes are altered also during human aging. Indeed, Lipinski et al. [77] found abnormal transcript levels of key autophagy mediators (Atg5 and Atg7) in the brain of older people [77]. Furthermore, reduced expression of microtubule-associated protein 1 light chain 3B (LC3B) has been retrieved in the vastus lateralis muscle of older hip-fractured patients with sarcopenia [78].

While senescence is associated with increased exosome secretion upon activation of p53 [79], EV concentration decreases during aging. Interestingly, EVs are taken up by B cells faster in older people than younger adults, suggesting that, during aging, circulating EV levels might be reduced as a result of enhanced internalization by immune cells [80].

Recent evidence indicates that EVs released by senescent cells may promote cancer cell proliferation [81] and vascular calcification [82], while reducing bone formation [83]. Increased secretion of EV-associated nuclear DNA by senescent cells has also been reported [84]. These genome fragments are pro-inflammatory and contribute to inflamm-aging [85]. Circulating levels of mtDNA molecules increase progressively past the age of 50 and have been associated with chronic low-grade inflammation [86,87]. Indeed, exposure of monocytes to mtDNA concentrations similar to those detected in vivo induces tumor necrosis factor-α production [86]. In neutrophils, blockade of TFAM-mediated rerouting of oxidized mtDNA to lysosomes is followed by extrusion of oxidized nucleoids that eventually trigger interferon I activation [88]. However, whether cell-free circulating mtDNA is contained within EVs is currently unclear.

An additional link between mitochondrial dysfunction and systemic inflammation is mediated by the nucleotide-binding and leucine-rich repeat receptor (NLR) family pyrin domain containing 3 (NLRP3) inflammasome [89]. NLRP3 inflammasome activity is blunted by autophagy [90] and stimulated by mitochondrial-generated reactive oxygen species (ROS) [91]. In turn, NLRP3-dependent engagement of caspase-1 blocks mitophagy, thereby reducing the clearance of ROS-producing damaged mitochondria and, hence, reinforcing inflammasome activation [92]. In the context of sustained defective mitophagy, the accrual of dysfunctional mitochondria may eventually result in the extrusion of MDVs stimulating an inflammatory response. Conversely, when autophagy is fully working NLRP3-induced mtDNA release into the cytosol is inhibited, which mitigates inflammation [93].

Dysfunctional mitochondrial–lysosomal axis in conjunction with abnormal EV trafficking has recently been involved in the pathogenesis of neurodegenerative diseases, including AD [94], Parkinson’s disease [95], and Huntington disease [96]. During neurodegeneration, mitochondrial dysfunction resulting from defective mitophagy contributes to synaptic disruption and neuronal loss by promoting accumulation of misfolded proteins (e.g., amyloid β, hungtintin, tau, alpha-synuclein) through increased oxidative damage and impaired bioenergetics [97]. In such a context, EV trafficking may have neuroprotective or detrimental effects depending on the origin and composition of vesicles. Indeed, MVBs can follow two alternative fates: some are delivered to lysosomes for degradation, therefore promoting clearance of cellular waste, while others fuse with the plasma membrane to release ILVs into the extracellular space as exosomes [94]. This second route may allow delivery of misfolded and harmful proteins to neighboring cells through a prion-like mechanism, thereby contributing to disease progression [98].

The broad spectrum of molecules packaged within exosomes and secreted into body fluids makes them valuable biomarkers for identifying and tracking several disease conditions. Furthermore, the characterization of exosomal pathways holds promise as a platform for developing novel therapeutic interventions. Indeed, due to their ability to cross the blood–brain barrier, exosomes might be used to deliver drugs directly into the central nervous system [99,100]. The low immunogenic potential of exosomes and their prion-like behavior also render these vesicles ideal nanodelivery systems for RNAi therapy [101] and immunotherapy [102]. Eventually, technological advancements in biofluidics and informatics may allow EVs to be harnessed as "liquid biopsies" for personalized medicine and delivery systems for precision medicine [103]. 

## 5. Conclusions

Substantial evidence supports the existence of a mitochondrial–lysosomal axis acting in synergy with other cellular quality control systems. The age-related breach in general and selective autophagy impairs the removal of damaged cellular components, including dysfunctional mitochondria, that therefore need to be disposed of otherwise. In such a context, mitochondria may feed EV trafficking via an endolysosomal delivery system. Disruption of the mitochondrial–lysosomal axis coupled with abnormal EV secretion has been implicated as a mechanism contributing to the aging process as well as to a wide range of disease conditions. However, the knowledge of the mechanisms responsible for the coordination between EV trafficking and the mitochondrial–lysosomal axis is still fuzzy. Understanding the molecular actors involved in such a process may eventually allow developing innovative anti-aging remedies and personalized interventions against disease conditions associated with altered cellular quality control.

## Abbreviations

AD	Alzheimer’s disease
AβOs	Amyloid-β oligomers
AIF	Apoptosis inducing factor
Atg	Autophagy-related gene
DRP1	Dynamin-related protein 1
ESCRT	Endosomal sorting complex required for transport
EV	Extracellular vesicle
FIS1	Mitochondrial fission 1 protein
ILV	Intraluminal vesicle
LC3	Microtubule-associated protein 1A/1B-light chain 3
MDV	Mitochondrial-derived vesicle
MFN	Mitofusin
MQC	Mitochondrial quality control
mtDNA	Mitochondrial DNA
mTORC1	Lysosomal mechanistic target of rapamycin complex 1
MVBs	Multivesicular bodies
NiMA	Nutrient-induced Mitochondrial Activity
NLRP3	Nucleotide-binding and leucine-rich repeat receptor (NLR) family pyrin domain containing 3
OPA1	Optic atrophy-1
PINK1	Phosphatase and tensin homolog (PTEN)-induced putative kinase 1
RAB	Ras-related protein in Brain
RAL1	Ras Like-1
ROS	Reactive oxygen species
SASP	Senescence-associated secretory phenotype
TFAM	Mitochondrial transcription factor A
